# Knockout of 5-Lipoxygenase Results in Age-Dependent Anxiety-Like Behavior in Female Mice

**DOI:** 10.1371/journal.pone.0029448

**Published:** 2011-12-29

**Authors:** Yash B. Joshi, Domenico Praticò

**Affiliations:** Department of Pharmacology, Temple University School of Medicine, Philadelphia, Pennsylvania, United States of America; University of Illinois-Chicago, United States of America

## Abstract

**Background:**

The enzyme 5-lipoxygenase (5LO) has been implicated in a variety of neurological and psychiatric disorders including anxiety. Knockout of 5LO has previously been shown to alter anxiety-like behavior in mice at a young age but the effect of 5LO knockout on older animals has not been characterized.

**Methodology/Principal Findings:**

Here we used the elevated plus maze behavioral paradigm to measure anxiety-like behavior in female mice lacking 5LO (5LO-KO) at three different ages. Adolescent 5LO-KO animals did not significantly differ from wild-type (WT) animals in anxiety-like behavior. However, adult and older mice exhibited increased anxiety-like behavior compared to WT controls.

**Conclusions:**

These results indicate that 5LO plays a role in the development of the anxiety-like phenotype in an age-dependent manner in female mice. Future work should further investigate this interaction as 5LO may prove to be an important molecular target for the development of novel anxiolytic therapies.

## Introduction

5-lipoxygenase (5LO) is an enzyme that converts arachidonic acid to leukotriene metabolites, fatty acid signaling molecules that are important contributors to the pathophysiology of allergy, asthma and inflammation [1]–[3]. However, 5LO is also widely expressed in the central nervous system and emerging work suggests 5LO may play a role in neurological and psychiatric disorders. 5LO contributes to Alzheimer's disease pathology, prion protein toxicity as well as multiple sclerosis demyelination [4]–[6]. 5LO levels are altered in the brains of suicide victims and its pharmacologic inhibition has been shown to potentiate antidepressant activity in mice [7], [8]. 5LO has also been implicated as an important molecular target in anxiety, with knockout of 5LO (5LO-KO) reported to decrease anxiety-like behavior at a young age in male mice [9]. However, anxiety-like behavior in 5LO-KO mice has not been otherwise characterized. Clinical data indicates that anxiety disorders are more common in women and that lifetime prevalence of anxiety disorders changes with age, reaching a peak in adolescence to early adulthood [10]. 5LO is known to increase in an age-dependent manner in several brain regions and has been conjectured to serve a potential biological link between stressors and the development of neuropsychiatric illness [11]–[13]. Therefore, in this study we asked the question of what effect 5LO knockout (5LO-KO) has on anxiety-like behavior in mice at different ages. To this end, we used the elevated plus maze paradigm to compare the behaviors of wild-type (WT) and female 5LO-KO mice at 3, 6 and 12 months of age.

Our findings show that compared with WT, 5LO-KO mice manifest an age-dependent increase in anxiety-like behavior as measured by this paradigm.

Understanding the interactions between the 5LO pathway and the anxiety phenotype may provide the basis for a clinical therapeutic strategy in anxiety disorders and should be further investigated.

## Materials and Methods

### Animals

Female wild-type C57BL/6 (WT; The Jackson Laboratory) and 5LO knockout mice (5LO-KO; The Jackson Laboratory) aged 3, 6, and 12 months were used for this study. All mice were housed on a 12 hours light/dark cycle in the Medical Research Building at the Temple University Health Sciences Campus, which is fully accredited by the Association for the Assessment and Accreditation of Laboratory Animal Care International. Standard mouse chow and water were provided *ad libitum*. Animal procedures were conducted in accordance with the National Institute of Health guidelines for the use of experimental animals and were approved by the Temple University's Animal Care and Use Committee.

### Elevated plus maze

All animals were pre-handled for 3 days prior to testing. They were tested in a randomized order, and all tests were conducted by an experimenter blinded to the genotype. Anxiety-like behavior was assessed by using the elevated plus maze behavioral paradigm. The elevated plus maze (SD Instruments, San Diego, CA) utilized in this study was made of beige high density, non-porous plastic in the form of a plus sign with four arms of equal length (32 cm) and width (5 cm) elevated 40 cm off the ground. Two arms were closed off with 15.5-cm-high walls and two open arms had 0.5-cm-high ledges. Arms extended form a central square platform (5×5 cm). Room lighting was adjusted such that closed arm light levels were maintained at ∼160 lux and open arm light levels were maintained at approximately ∼200 lux. Each mouse was placed in center square facing a closed arm and was allowed to freely explore for 10 min while being video recorded. The elevated plus maze was cleaned with 30% ethanol and allowed to dry following each behavioral test. An entry was counted when the mouse had all four paws in an arm.

### Statistical analysis

Data are presented as the mean ± standard error of the mean. Percent of time spent in closed and open arms [(total time in arms/600 s) * 100] and percent of closed and open arm entries [(total arm entries/total entries) * 100] were calculated for each animal. The unpaired two-tailed student ­*t*-test with an alpha of P<0.05 was used to define significance.

## Results

### No effect on anxiety in adolescent mice lacking 5LO

**Figure 1:**
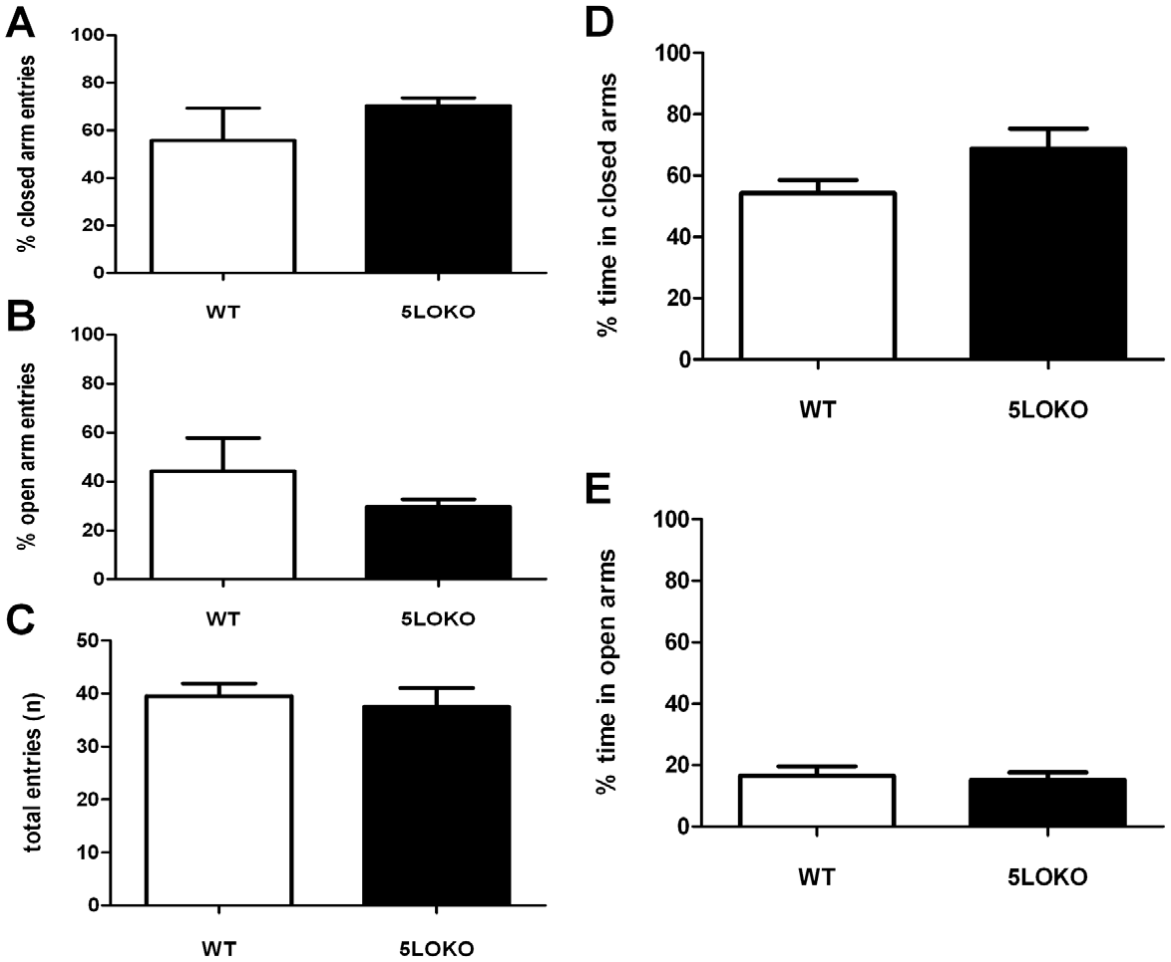
Elevated plus maze behavior in 3 month-old mice. Percentage of total entries into the closed arm (A), open arm (B), total arm entries (C), percentage of total time spent in closed (D) and open arm (E). No difference was detected in any parameter between wild type (WT) (open bars) and 5LO-KO mice (closed bars) (WT, n = 6; 5LO-KO n = 3).

First, we investigated whether 5LO knockout leads to changes in anxiety-like behavior in 3 month-old mice. As shown in Figure 1, no statistically significant differences were observed in the percentage of closed (WT: 55.7.2 %+12; 5LO-KO: 70%+3; P<0.2) or open arm entries (WT: 44.3%+12.2; 5LO-KO: 30%+3; P<0.2) between the two groups of mice. Similar results were observed when the total entries (WT: 39.5+2.1; 5LOKO: 38+4; P<0.6), and the percentage time spent in closed (WT: 54.3%+3.8; 5LO-KO: 68.8%+6.5; P<0.1) or open arms (WT: 16.6%+2.7; 5LO-KO: 15.2%+2.4; P<0.7) were evaluated (Fig. 1).

### Adult mice lacking 5LO exhibit greater anxiety-like behavior than wild type

**Figure 2:**
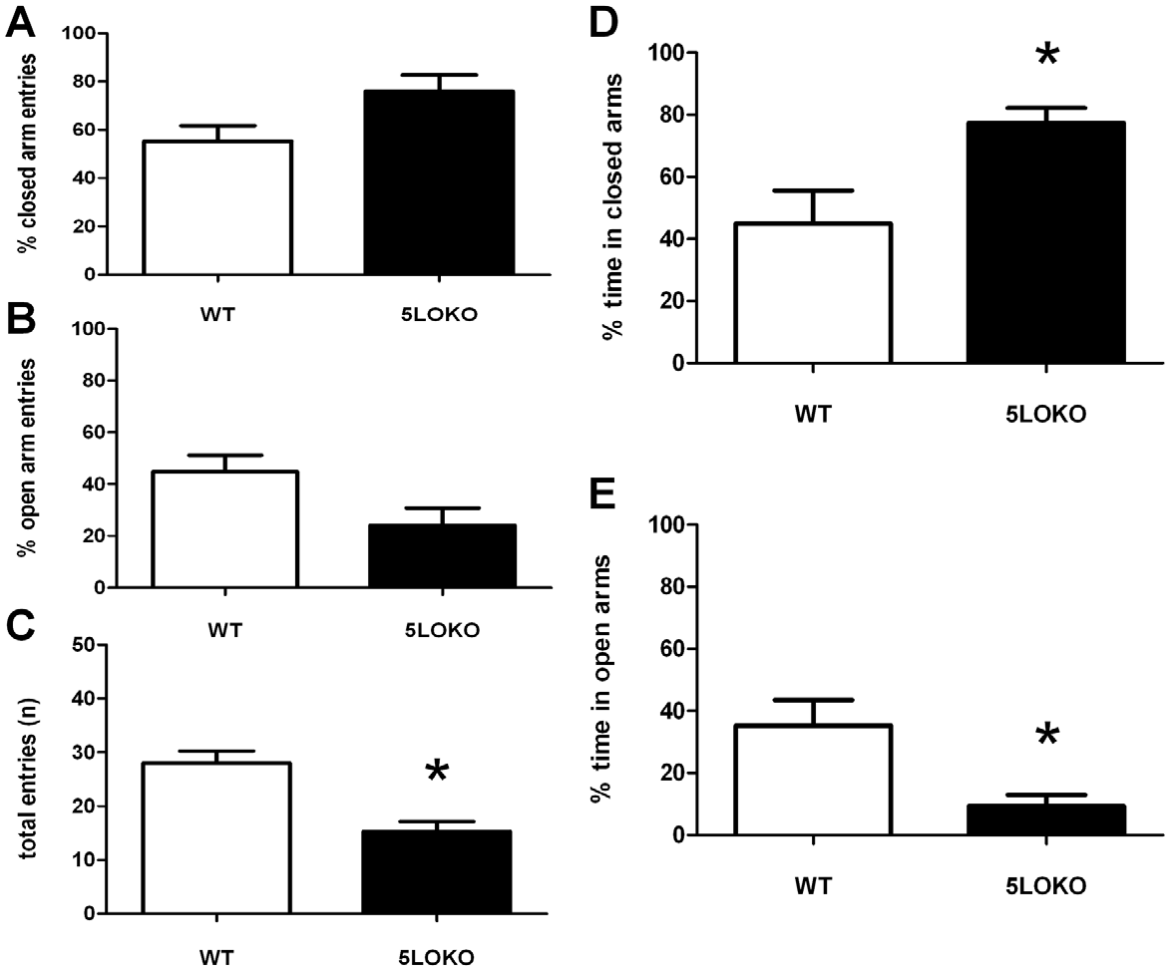
Elevated plus maze behavior in 6 mo old mice. Percentage of total entries into the closed arm (A), open arm (B), total arm entries (C) and percentage of total time spent in closed (D) and open arm (E). 5LO-KO mice (closed bars) spent more time in the closed arm and less time in the open arm than wild type (WT) mice (open bars). 5LO-KO animals also entered less total arms than WT. (WT, n = 4; 5LO-KO, n = 4; P<0.05).

Because anxiety disorders commonly develop in early and mid-adulthood, we asked whether 5LOKO mice at 6 months of age would display an anxiety-like phenotype different from adolescent mice. As shown in Figure 2, 5LO-KO mice spent statistically significant more time in the closed arms (WT: 44.8%+10.8; 5LO-KO: 77.3%+4.8; P<0.03), less time in the open arms (WT: 35.2%+8.3;5LO-KO: 9.4%+3.6; P<0.03), and had lower total number of entries (WT: 28+2.2;5LO-KO: 15.3+1.9; P<0.003). However, no significant difference in the percentage of entries into the closed (WT: 55.3%+6.4; 5LO-KO: 76.0%+6.8; P<0.06) or open arms (WT: 44.7%+6.4; 5LO-KO: 24.0%+6.8; P<0.06) was observed (Fig. 2).

### Mice lacking 5LO continue to exhibit anxiety-like behavior in late adulthood

**Figure 3:**
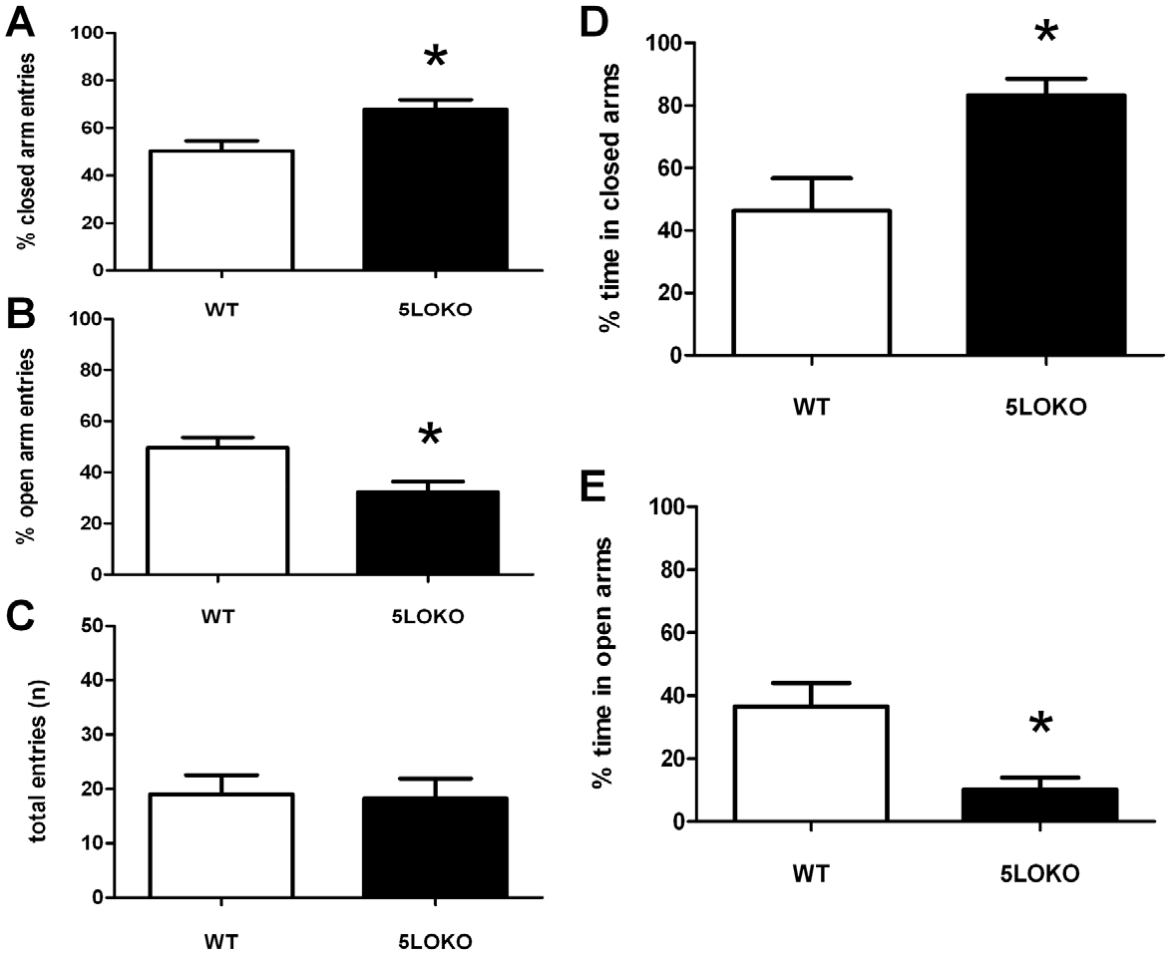
Elevated plus maze behavior in 12 mo old mice. Percentage of total entries into the closed arm (A), open arm (B), total arm entries (C) and percentage of total time spent in closed (D) and open arm (E). 5LO-KO mice (closed arms) entered the closed arms more and entered open arms less than wild type (WT) animals (open arms). 5LO-KO animals also spent more time in the closed arm and less time in the open arm than WT mice. No differences in total arm entries were noted. (WT, n = 4, 5LO-KO, n = 6).

Finally we investigated how the anxiety-like behavior is affected by 5LO knockout through late adulthood. As shown in Figure 3, 12-month-old 5LO-KO mice entered the closed arms significantly more (WT: 50.4 %+4.1; 5LO-KO: 67.7%+4.1: P<0.02) and the open arms significantly less (WT: 49.6%+4.1; 5LO-KO: 32.3%+4.1; P<0.02) than WT mice. Additionally, 5LO-KO mice spent significantly more time in the closed arms (WT: 46.3 %+10.4; 5LO-KO: 83.3%+5.1; P<0.02) and significantly less time in the open arms (WT: 36.5 %+7.57; 5LO-KO: 10.2% + 3.8; P<0.03). However, at this age no statistically significant difference was found in the number of total entries between genotypes (WT: 19 + 3.5; 5LOKO: 18.25 + 3.6: P<0.8) (Fig. 3).

## Discussion

The goal of the current study was to determine whether mice with the genetic absence of 5LO, which appear normal when left unchallenged, manifest modified anxiety-like behavior as measured by the elevated plus maze and whether there exists an age-dependent effect.

The elevated plus maze draws out competing preferences in mice between exploring novel environments (i.e., activity in open arms) and being in a protected environment (i.e., activity in closed arms). Low anxiety behavior is usually reflected by higher number of entries and/or length of time spent in open arms, while higher entries and/or time spent in closed arms corresponds to a pro-anxiety phenotype [14], [15].

In our study, we found that 5LO-KO animals did not differ from the WT cohorts at 3 months of age in any parameter of anxiety-like behavior as measured by this type of maze. However, at 6 months of age, differences in anxiety were apparent between groups as 5LO-KO spent more time in the closed arms and spent less time in the open arms than WT mice, reflecting a pro-anxiety behavior. By 12 months, 5LO-KO animals, in addition to spending greater time in the closed arm and less time in the open arms, also entered the closed arms more and entered the open arms less than WT animals.

Differences in total number of arm entries in the elevated plus maze as those we found at 6 months of age between WT and 5LO-KO animals may indicate that some locomotor differences exist between mice groups, which could potentially result in artifactual anxiety-like behavior. However, we believe that general locomotor differences are not the cause for the increased anxiety phenotype we observed in the 5LO-KO, as over time, total entries decreased in both WT mice and 5LO-KO mice from 39.5 and 37.5 at 3 months of age, to 19 and 18.5 at 12 months of age, respectively. This observation suggests that while the activity levels in both genotypes decline to similar levels over time, we sampled elevated plus maze behavior at an age where 5LO-KO mice had diminished locomotor activity but WT mice did not. Indeed, previous work demonstrates that there is an overall decline in locomotion as a function of aging in mice, and it has also been reported knockout of 5LO does not alter diurnal or nocturnal rodent activity [16], [17]. Importantly, the significant differences we observed in 5LO-KO at 6 months in percentage of time spent in closed and open arm were reproduced at 12 months of age.

Taken together, our findings support the new hypothesis that in the central nervous system the 5LO products of the arachidonic acid metabolism (i.e, 5-HETEs, leukotrienes), among other activities, may have an inhibitory action on mechanisms involved in the development of anxiety-like symptoms.

The observation that 5LO-KO leads to an age-dependent increase in anxiety-like behavior is in contrast with a previous paper where a reduction in this type of behavior was reported [9]. In their study, the authors used mice aged 2–3 months and found significant increases in open arm entries and time spent, while also finding reduction in time spent in the closed arms. Though we found no differences in elevated plus maze anxiety-like behavior between WT and 5LO-KO mice aged 3 months, this observation could be because Uz and colleagues used male mice in their behavioral experiments while in our study we used female mice. It is possible that a difference in gender might account for such a discrepancy. It has been shown that the production of leukotrienes, the downstream metabolites of 5LO, is substantially lower in whole blood and neutrophils in human males compared to females, which has been shown to result from testosterone-influenced differences in sub-cellular localization of 5-LO [18], [19]. Such a difference in 5LO efficiency is also thought to underlie gender-based differences in the therapeutic response of 5LO inhibitors [20]. Therefore, the genetic absence of 5LO may alter downstream signaling pathways differently in male and female mice.

The fact that the anxiety-like behavior increased in adulthood and at an age thought to represent late adulthood or early senescence in mice, is particularly intriguing given that we found no difference in the adolescent mice. Thus, it is possible to speculate that the permanent absence of 5LO metabolites in these mice influences brain circuitry which, while not observable at a young age, manifests in adulthood and is responsible for the altered phenotype. Interestingly, a polymorphism in the 5LO gene has been described that influences endogenous 5LO content and results in significant pharmacogenetic implications [21]. Recent work has also implicated different polymorphisms in a downstream enzyme in the 5LO pathway that may be protective against depression, but only in women [22]. Furthermore, 5LO pathway inhibitors, while generally well tolerated, have been recently shown to have adverse side-effects including an increase in anxiousness in post-market surveillance. Given this emerging evidence, it is possible that an interaction between gender and 5LO and 5LO pathway polymorphisms could alter an individual's susceptibility to anxiety behavior.

In conclusion, our work demonstrates that knockout of 5LO leads to an age-dependent increase in anxiety-like behavior in female mice. Future work must further investigate the mechanism of these behavioral alterations and establish the role of 5LO in mediating the anxiety phenotype. Successful accomplishment of this task could lead to novel therapeutic strategies aimed at mitigating the symptoms of anxiety disorders.
